# Tongue Manifestation in Patients with Osteonecrosis of the Femoral Head: A Cross‐sectional Study

**DOI:** 10.1111/os.13388

**Published:** 2022-07-27

**Authors:** Yan Jia, Jigao Sun, Zhaoxu Jia, Zhipeng Xue, Rongtian Wang, Haijun He, Weiheng Chen

**Affiliations:** ^1^ Department of Minimally Invasive Arthrology The Third Affiliated Hospital of Beijing University of Chinese medicine Beijing China; ^2^ Department of Orthopedics Dongfang Hospital Beijing University of Chinese Medicine Beijing China; ^3^ Department of Orthopedics Fangshan Hospital Beijing University of Chinese Medicine Beijing China; ^4^ Third Department of Orthopedics Wangjing Hospital, China Academy of Chinese Medical Sciences Beijing China

**Keywords:** Cross‐sectional study, Osteonecrosis of the femoral head, Tongue manifestation, Traditional chinese medicine

## Abstract

**Objective:**

Although tongue manifestation is a vital component of Traditional Chinese Medicine (TCM), relevant research on patients with osteonecrosis of the femoral head (ONFH) is still lacking. This study will explore the characteristic tongue manifestation of ONFH patients to inform future research and clinical practice.

**Methods:**

This is a cross‐sectional study. All ONFH patients meeting criteria and their clinical data were collected from the online China osteonecrosis of the femoral head database (CONFHD) since it was created. Organized tongue manifestations of eligible patients through the tongue manifestation acquisition instrument, including tongue shape, tongue color, tongue coating thickness, tongue coating color and tongue coating moisture. We used descriptive analysis for the general information while systematic clustering analysis for the better summary of tongue characteristics.

**Results:**

A total of 375 ONFH patients were included with an average age of 46.3 years. Most patients appeared with enlarged tongue body (54.4%), and the proportions of pale and red tongue (62.4%) were higher than others. Tongue coating were mainly showed as thick (64.5%), white (57.6%) and moist (79.7%). Comparison of tongue shape between different causes of ONFH had a significant statistically difference (*P* = 0.000). Tongue manifestations could be cluster analyzed into three categories which were matched into four TCM syndromes.

**Conclusions:**

The tongue manifestation of ONFH patients has a significant change both in tongue body and coating, and different features may be related to the ONFH pathology. This study provides new and valuable tongue informations for a preliminary screening of ONFH patients.

## Graphical abstract

The tongue manifestation of ONFH patients has a significant change both in tongue body and coating. They could be cluster analyzed into three categories which were matched into four TCM syndromes, and different features may be related to the ONFH pathology.

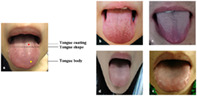

## Introduction

Osteonecrosis of the femoral head (ONFH) is a progressive orthopedic disease that mainly affects young and middle‐aged adults, and can cause lifelong disability.^1^ More than 8 million people suffer from non‐traumatic ONFH in China.^2^ Because of its social and economic burden, many surgeons attempt to delay performing total hip arthroplasty when making decisions.^3^, ^4^ While ONFH has become a serious challenge of public health, there is still a lack of standard treatment regimen to slow the disease progression currently.^5^, ^6^ In fact, several studies have shown that Traditional Chinese Medicine (TCM) can improve the quality of life in ONFH patients.^6^, ^7^, ^8^, ^9^

Tongue manifestation used as an important diagnostic skill is the vital component of the TCM diagnosis system to examine the disease conditions and pathological changes, which has been repeatedly verified by clinical TCM practitioners for more than 2000 years owing to its simple, non‐invasive and valuable diagnostic procedure.^10^, ^11^ It is based on the tongue feature relating to the changes in size, shape, color and moisture of the tongue body and coating. Tongue body means the musculature and vascular tissue. Tongue coating is defined as a layer of moss‐like material covering the tongue that consists of epithelium, saliva, microorganism, food debris, and constitutes a large surface area for microbes shedding and cellular desquamation.^12^, ^13^ According to TCM theory, the biggest role of the tongue manifestation is to reflect whether organ functions or basic substances (such as *Qi*, blood) keep balance in human body.^14^, ^15^ *Qi* refers both to the refined nutritive substance that flows within the human body as well as to its functional activities. It is the basic element that, through its movements and transformations, produces the human life activities. Abnormal colors and shapes suggest unhealthy body states, it is useful for judging the disease process, severity and therapeutic effect by the evolution of overall tongue manifestations. For example, the unusual pale red tongue with some purple petechiae and white thick coating means blood stasis or a lack of *Qi* and blood occurs in those who have been long‐term disease, if the treatment is effective, both color and shape of tongue will return to normal; otherwise the opposite will happen. Modern research found there were certain correlations between tongue manifestation and biochemical examination, microcirculation, blood cells and tissue fluid, etc.^16^ Therefore, beyond serving as an early diagnosis tool, tongue manifestations also provide evaluation indicators for clinical differential diagnosis and treatment of TCM.^12^, ^17^, ^18^, ^19^

With the combination of various technologies and tongue diagnosis,^11^, ^20^, ^21^ the modern exploration of tongue manifestation is widely occurring in clinical practice of many diseases like eczema, diabetes, rheumatoid arthritis and cancer.^11^, ^21^, ^22^, ^23^, ^24^, ^25^ A single‐center study published by our research team in 2015, involving 273 non‐traumatic ONFH patients, found that there were significant differences in their characteristics of the tongue at different ARCO phases.^26^ Through cluster analysis, it divided tongue manifestation into three categories: at ARCO phase I, most tongues were pale purple and enlarged with thin coating; at ARCO phase II and III, most tongues were red or purple and enlarged with white coating; and at ARCO phase IV, most tongues were dark red, enlarged and teeth‐marked with yellow thin coating. But to the best of our knowledge, no similar study on etiology of ONFH with a larger sample and multiple centers has been published yet. Thus, we attempted to make a objective preliminary exploration on ONFH patients by using an advanced tongue manifestation acquisition instrument, and aimed to: (i) summarize tongue manifestation of ONFH patients; (ii) explore the relationship between tongue manifestation and etiology of ONFH; and (iii) better inform future research and clinical practice.

## Methods

### Data Sources

The clinical data were acquired from the China osteonecrosis of the femoral head database (CONFHD, http://onfh.keyanyun.com/). CONFHD is a Chinese online platform focused on ONFH that provides ambulatory care, inpatient care, management, and pharmaceutical data. General information such as age, gender, causes, TCM syndrome type, Association Research Circulation Osseous (ARCO) stage, imaging data, treatment methods, Harris hip score, visual analogue scale (VAS) score should be kept on file. Between July 2016 and December 2018, 25 hospitals from 12 provinces (autonomous areas or municipalities) took part in the development and collecting of cases for CONFHD. This study has been approved by the hospital ethics committee (No. 2008KT15).

### Inclusion and Exclusion Criteria

Inclusion criteria: all of the patients had been clearly diagnosed with ONFH by radiological examination (X‐ray, MRI or CT); complete tongue manifestation records or clear tongue image input. The following categories were excluded: (i) patients also suffered from serious physical diseases such as tumor, organ failure; (ii) children under the age of 18; (iii) pregnant or lactating women; (iv) have undergone surgical treatment; and (v) inadequate data such as missing age, sex, or causes.

### Definition and Classification

The WHO worldwide standard terminologies were applied to identify the terminology used in this investigation.^27^ Set the classification system of collected tongue manifestations according to standard TCM diagnostic principles.^28^ Tongue manifestation information includes tongue shape, tongue color, tongue coating thickness, tongue coating color and tongue coating moisture. The categorization technique is determined ahead of time for analysis (Table 1):

1. Tongue shape: the shape of tongue body which are mainly muscularture and vascular. The enlarged tongue become larger than normal in transverse diameter, usually bearing dental indentations on the margin. The thin tongue is thinner than normal.
2. Tongue color: the color of tongue body often appears in pale, pale red, red, crimson, or purple. Petechia refers to red or purple spots on the tongue body. The normal color of tongue is pale red.
3. Tongue coating thickness: tongue coating is a layer of moss‐like material covering, also called tongue fur. A thin coating means that the underlying tongue surface is faintly visible through it, but a thick coating cannot.
4. Tongue coating color: the color of tongue coating often appears in white or yellow.
5. Tongue coating moisture: the moisture of tongue coating often appears in moist or dry. A dry coating lacks moisture so it looks dry, but a moist coating is the opposite.

**TABLE 1:** Classification of tongue diagnosis

Tongue manifestation	Design and classification
Tongue body shape	Normal, Enlarged, Thin
Tongue body color	Pale, Pale red, Red, Crimson, Purple, Petechia
Tongue coating thickness	Thin, Thick
Tongue coating color	White, Yellow
Tongue coating moisture	Moist, Dry

### Data Extraction

Two researchers (ZPX and ZXJ) screened the CONFHD cases rigorously following the inclusion and exclusion criteria, extracting the general data such as age, sex, etiology, ARCO stage, and tongue features. For bilateral patients, the heavier side shall prevail. The DS01‐B tongue manifestation acquisition instrument was used to organize and evaluate all of the tongue pictures (Daosh Co., Shanghai, China), which is made up of a photography system and a software analysis system that can automatically measure tongue manifestation features. Previous research have shown that this tool is helpful in analyzing tongue features.^24^, ^29^

### Statistical Analyses

SPSS statistical software was used to handle and analyze all of the collected data (version SPSS 25.0, Chicago, IL, USA). The overall information was described using descriptive analysis, and the categorized variables were reported as percentages. The chi‐square test was used to determine whether or not there were significant differences between groups, and the *Post hoc* test was used for pairwise comparison. *P* value <0.05 was deemed of statistical significance. In addition, we used a systematic clustering analysis approach to summarize and categorize tongue features more standardly, and variables with a component ratio of less than 5% were eliminated during screening. This system would adequately judge whether items belong to one same category based on the homogeneity of the observation objects,^30^ and then the tongue manifestation information was matched with the TCM symptoms differentiation of ONFH.

## Results

### General Information

It was a cross‐sectional research, with 375 individuals eventually recruited with complete tongue data. The average age of the participants was 46.3 ± 12.3 years, with men accounting for 73.6% of the total. Table 2 summarizes the characteristics of the patients who were included in the study.

**TABLE 2:** Characteristics of 375 ONFH patients

Variables	Number	Percent (%)
Sex		
Male	276	73.6
Female	99	26.4
Age group		
18–39	119	31.7
40–59	201	53.6
≥60	55	14.7
ARCO stage		
I	2	0.5
II	109	29.1
III	175	46.7
IV	89	23.7
Etiology		
Idiopathic	177	47.2
Alcoholic	138	36.8
Corticosteroid	44	11.7
Trauma	16	4.3
Harris score		
≥70	76	20.3
<70	299	79.7
VAS score		
≥6	169	45.1
<6	206	54.9

### Tongue Manifestation

The characteristics of tongue manifestation are summarized in Table 3. The tongue shape of 375 ONFH patients was mainly enlarged (54.4%), the proportions of pale or red tongue color (62.4%) were higher than other tongue colors. Tongue thick coating was more than the thin (64.5% *vs*. 35.5%), coating color was mainly white (57.6%), and tongue moist coating took the majority (79.7%). Subgroup analysis excluded the influence of gender and age on most tongue manifestations, with the exception of specific tongue shapes between age groups (Table 4).

**TABLE 3:** Tongue manifestation of 375 ONFH patients

Tongue manifestation	Number	Percent	*P* value
Tongue body shape			
Normal	121	32.3	
Enlarged	50	13.3	0.000
Thin	204	54.4	
Tongue body color			
Pale	120	32.0	
Pale red	67	17.9	0.000
Red	114	30.4	
Crimson	33	8.8	
Purple	38	10.1	
Petechia	3	0.8	
Tongue coating thickness			
Thin	133	35.5	0.000
Thick	242	64.5	
Tongue coating color			
White	216	57.6	0.003
Yellow	159	42.4	
Tongue coating moisture			
Moist	299	79.7	0.000
Dry	76	20.3	

**TABLE 4:** Gender and age analysis of tongue manifestation (n, %)

Tongue manifestation	Tongue body shape			Tongue body color						Tongue coating thickness		Tongue coating color		Tongue coating moisture	
	Normal	Enlarged	Thin	Pale	Pale red	Red	Crimson	Purple	Petechia	Thin	Thick	White	Yellow	Moist	Dry
Sex															
Male	92 (76.0)	36 (72.0)	148 (72.5)	95 (79.2)	49 (73.1)	82 (71.9)	23 (69.7)	24 (63.2)	3 (100.0)	92 (69.2)	184 (76.0)	159 (73.6)	117 (73.6)	223 (74.6)	53 (69.7)
Female	29 (24.0)	14 (28.0)	204 (27.5)	25 (20.8)	18 (26.9)	32 (28.1)	10 (30.3)	14 (36.8)	0	41 (30.8)	58 (24.0)	57 (26.4)	42 (26.4)	76 (25.4)	23 (30.3)
χ^2^	0.55			5.165						2.079		0		0.732	
*p*	0.759			0.377						0.149		0.995		0.392	
Age group															
18–39	46 (38.0)	9 (18.0)	64 (31.4)	42 (35.0)	22 (32.8)	32 (28.1)	12 (36.4)	11 (28.9)	0	42 (31.6)	77 (31.8)	66 (30.6)	53 (33.3)	94 (31.4)	25 (32.9)
40–59	64 (52.9)	28 (56.0)	109 (53.4)	63 (52.5)	32 (47.8)	65 (57.0)	16 (48.5)	22 (57.9)	3 (100.0)	72 (54.1)	129 (53.3)	119 (55.1)	82 (51.6)	160 (53.5)	41 (53.9)
≥60	11 (9.1)	13 (26.0)	31 (15.2)	15 (12.5)	13 (19.4)	17 (14.9)	5 (15.2)	5 (13.2)	0	19 (14.3)	36 (14.9)	31 (14.4)	24 (15.1)	45 (15.1)	10 (13.2)
χ^2^	11.534			5.37						0.033		0.469		0.191	
*p*	0.021			0.872						0.984		0.791		0.909	

### Relationship between Tongue Manifestation and Etiology

The comparison of tongue shape with different causes of ONFH revealed a statistically significant difference (*P* = 0.000). Further investigation indicated that alcoholic ONFH patients have a lower proportion of thin tongue than patients with other causes (Adjusted R = −5.8). There were no statistically significant differences in tongue coating characteristics and tongue colors caused by various factors (Tables 5 and 6).

**TABLE 5:** Comparison of tongue body and tongue color with different causes (n, %)

Tongue manifestation	Trauma	Alcoholic	Corticosteroid	Idiopathic	*P* value	χ^2^ value
Tongue body shape						
Normal	3 (18.8)	54 (39.1)	19 (43.2)	45 (25.4)	0.000	64.078
Enlarged	10 (62.5)	84 (60.9)	24 (54.6)	86 (48.6)		
Thin	3 (18.8)	0 (0.0)	1 (2.3)	46 (26.0)		
Tongue body color						
Pale	6 (37.5)	53 (38.4)	7 (15.9)	54 (30.5)		
Pale red	2 (12.5)	27 (19.6)	6 (13.6)	32 (18.1)	0.120	20.266
Red	6 (37.5)	33 (23.9)	20 (45.5)	55 (31.1)		
Crimson	0 (0.0)	12 (8.7)	6 (13.6)	15 (8.5)		
Purple	2 (12.5)	12 (8.7)	5 (11.4)	19 (10.7)		
Petechia	0 (0.0)	1 (0.7)	0 (0.0)	2 (1.1)		

**TABLE 6:** Comparison of tongue coating with different causes (n, %)

Tongue manifestation	Trauma	Alcoholic	Corticosteroid	Idiopathic	*P* value	χ^2^ value
Tongue coating thickness						
Thick	12 (75.0)	91 (65.9)	25 (56.8)	114 (64.4)	0.556	2.031
Thin	4 (25.0)	47 (34.1)	19 (43.2)	63 (35.6)		
Tongue coating color						
White	8 (50.0)	79 (57.3)	26 (59.1)	103 (58.2)	0.930	0.451
Yellow	8 (50.0)	59 (42.8)	18 (40.9)	74 (41.8)		
Tongue coating moisture						
Moist	15 (93.8)	111 (80.4)	33 (75.0)	140 (79.1)	0.478	2.490
Dry	1 (6.3)	27 (19.6)	11 (25.0)	37 (20.9)		

### Tongue Manifestation Cluster Analysis

One tongue color item (Petechia) was eliminated since it accounted for less than 5% of the total, and the remaining 14 items were subjected to cluster analysis. When the scale was set as 20, 14 objects had better dispersion and could be categorized into three groups.

**Fig. 1:**
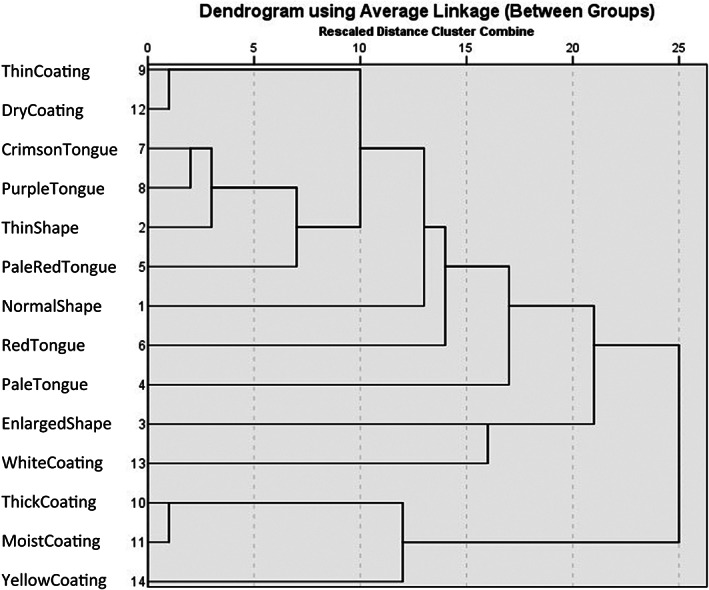
Three categories of tongue manifestation clustering analysis when the rescaled distance was set as 20. Category 1 (9 items): normal or thin tongue shape; pale, pale red, red, crimson or purple tongue color; thin and dry tongue coating. Category 2 (2 items): enlarged tongue shape; white tongue coating. Category 3 (3 items): thick, moist and yellow tongue coating

Three groups of tongue manifestations were categorized on experts judgments and clinical experience (Fig. 1). Category 1: normal or thin tongue shape; pale, pale red, red, crimson or purple tongue color; thin and dry tongue coating. Category 2: enlarged tongue shape; white tongue coating. Category 3: thick, moist and yellow tongue coating.

### Relationship between Tongue Manifestation and TCM Syndrome of ONFH

**Fig. 2:**
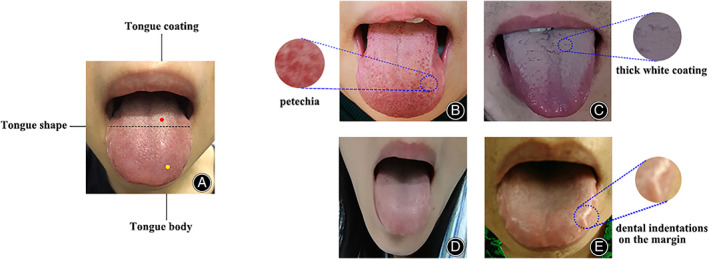
(A) Normal tongue is shown as pale red tongue and thin white coating. Red point stands for tongue coating; yellow point stands for tongue body; black dotted line represents the tongue body shape relative to mouth. (B) TCM type I: red tongue, thin coating with petechia. A 48‐year‐old male patient with idiopathic ONFH, ARCO stage III, Harris score 69. (C) TCM type II: purple tongue, thick white coating. A 37‐year‐old male patient with alcoholic ONFH, ARCO stage III, Harris score 69. (D) TCM type III: pale tongue. A 38‐year‐old female with corticosteroid ONFH, ARCO stage II, Harris score 58. (E) TCM type IV: enlarged tongue bearing dental indentations on the margin, moist white coating. A 54‐year‐old female patient with idiopathic ONFH, ARCO stage IV, Harris score 65.

We compared tongue manifestation clustering analysis findings with the TCM syndrome for ONFH guideline;^31^, ^32^ three categories of tongue manifestation could be matched into four kinds of tongue diagnostic types, which were connected to TCM syndrome for ONFH (Table 7). Figure 2 depicts representative tongue images of the four TCM syndrome kinds.

**TABLE 7:** Correspondence between tongue manifestation clustering analysis and TCM Syndrome of ONFH

Tongue manifestation clustering analysis	TCM syndrome type	Pathogenesis and pathological implications
Category 1 (1) Normal or thin, crimson or purple tongue, thin and dry tongue coating	Type I: *Qi* stagnation and blood stasis Type II: meridian obstruction	Hip trauma injuries local blood network, resulting in the blocking of blood circulation in the femoral head. Or *Qi* and blood are unbalance or stagnant, causing abnormal function of meridians.
(2) pale, pale red or red tongue	Type III: deficiency of the liver and kidney	Insufficient *Qi* and blood, deficiency of viscera function, leading to long‐term lack of nutrients for the femoral head.
Category 2 Enlarged tongue body, white coating	Type IV: phlegm‐stasis blocking collateral	The substances produced by alcohol, corticosteroid or other issues affect the blood vessels in the femoral head.
Category 3 Thick moist yellow tongue coating		The produced substances block the local blood vessels for a long time, leading to the changes in the metabolism of body fluids.

Category 1: (i) normal or thin, crimson or purple tongue, and thin or dry tongue coating are consistent with the meridian obstruction, *Qi* stagnation and blood stasis; (ii) pale, pale red or red tongue are consistent with the deficiency of the liver and kidney. For categories (ii) and (iii), the enlarged tongue body with white coating and the thick moist yellow tongue coating always emerge in phlegm‐stasis blocking collateral.

## Discussion

This study conducted a preliminary research on the tongue characteristics of ONFH patients using DS01‐B tongue manifestation acquisition instrument, and investigated the relation between tongue manifestation and etiology. Three tongue manifestation categories divided through cluster analysis may disclose the early, middle and late stage of ONFH when associated with TCM syndrome differentiation.

### Chinese Medicine Interpretation of Tongue with ONFH

ONFH symptoms are referred to in TCM as “Gushi” or “Bi syndrome,” which corresponds to bone erosion and obstruction. It has a variety of causes, however the etiology and pathology are yet unknown. Increased interosseous pressure, lipid metabolic disorder, intravascular coagulation, and osteoporosis, among other theories, have been proposed by several scientists. What has been confirmed is that the disruption of blood supplies to the femoral head is the main mechanism of osteonecrosis.^33^, ^34^, ^35^ Whether it is trauma, alcohol or hormones, according to latest researches, these factors tend to cause local vascular blockage in the femoral head as well as unbalanced *Qi* and blood.^36^, ^37^, ^38^ Tongue and its coating are always influenced by the blood circulation, lymphatic system, and tongue epithelial cell metabolism. As the basic diagnosis theories of TCM said, changes in tongue shape and color appear to suggest an imbalance of *Yin* or *Yang*, *Qi* or blood, which are equivalent to the human body's condition and objective evidence of disease.^25^ Therefore we may get useful information about the blood supply of the femoral head through tongue manifestation.^39^, ^40^

### Typical Tongue Manifestation of ONFH Patients

The typical normal tongue manifestation is characterized by a moderate tongue shape, pale red tongue body, and a thin white tongue coating. A previous study^41^, ^42^ involving 5403 healthy adults showed that 68.20% of them had moderate tongue shape, 81.82% had pale red tongue color, and 75.88% were thin white tongue coating, which is evidence of being normal. Although no studies have proven the tongue manifestation is related to gender, age, or race at present, clinicians agree that physical constitution and living climate may result in physiological variations, which can generate some non‐pathological differences between infants and the elderly, southerners and northerners. The enlarged or thick often represents the exuberance whereas the thin usually represents the deficiency. Based on CONFHD, we discovered that the tongue manifestation of ONFH patients was aberrant if compared with healthy person, which typically showed as enlarged tongue body in pale or red, and a moist thick white tongue coating. Tongue manifestation clustering analysis results correspond to TCM syndrome and follow their pathogenesis theories of differentiation.

The enlarged, pale or red tongue body of ONFH patients indicates abnormal tongue microcirculation. The red tongue is related to dilation of peripheral capillaries and increased high‐speed tongue blood flow, patients may have blood stasis either. On the contrary, it appears as a pale tongue when the decrease in red blood cell quantity, protein insufficiency, tissue edema, and insufficient blood flow occur, which is also common in patients with anemia. The 6‐keto‐prostaglandin in plasma, amounts of fungiform papillae, levels of thromboxane B2 and T‐lymphocytic subsets CD4 + can all be utilized to make a diagnosis of pale tongue color.^16^, ^43^ Referring to one study on the chemical markers of tongue coating epithelium, LDH, MDH, and ACP of white tongue coating were lower than the normal, indicating the growth, differentiation, dissolution and degradation of cells were relatively slow, and the level of intracellular oxidation was low.^44^ Several studies revealed there were significant changes in hemorheology in patients with a thick tongue coating. The cells in the keratodermis will not fully keratosis or fall off on time due to aberrant metabolism, resulting in an unnatural thick tongue coating when linked to the surface of the tongue back. Furthermore, smoking and drinking can thicken the tongue coating.^45^, ^46^, ^47^, ^48^ The previous clinical analysis of our group also found that non‐traumatic ONFH has significant intravascular lipid and coagulation abnormalities.^33^ In this study, alcoholic ONFH patients accounted for the highest proportion of thick coating, reaching 65.9%.

### Different Etiology of ONFH May Have Different Tongue Shape

Meanwhile, we analyzed the tongue characteristics of ONFH between different causes, but only the shape of the tongue differed significantly across the different causes of ONFH. Further analysis revealed that an enlarged tongue was shown to be more common in alcoholic and idiopathic ONFH patients; subgroup analysis also suggests that tongue body shape were specific between different age groups. One study found differences in tongue body shape between two VAS groups (VAS <6 *vs*. VAS ≧6).^49^ Thus, tongue body shape is influenced by a variety of factors, but the pattern is not clear yet. Alcohol or other factors might cause abnormal lipid distribution, which can restrict the local blood supply.^50^ No statistically significant difference in tongue color or tongue coating has been revealed between the various sources of ONFH.

Our study has some limitations. First, we did not limit the inclusion and exclusion criteria very strictly. Some participants may have some comorbidities such as hypertension, osteoarthritis, diabetes, etc., and these comorbidities may have an influence on the tongue manifestation. Second, this study does not emphasize a unified evaluation point. CONFHD records the tongue manifestations of patients at their first visit in the hospital, but some of them have tried some treatment in other healthcare institutions or clinics outside the 25 hospitals included in this article, those ineffective treatment experiences might have little impact on this study. Third, the specific characteristics of ONFH patients with diverse etiology and the degree of crushing are currently being investigated. Fourth, we just ran a cluster analysis on the collected tongue manifestations, but the changing law throughout the course of ONFH remained a mystery. Despite the fact that our study revealed tongue manifestation features in ONFH patients, it may not encompass all types of tongue feature alterations in ONFH patients, and the therapeutic usefulness and possible advantages of tongue diagnosis remain to be determined.

### Conclusions

In conclusion, this study suggested the typical tongue manifestation of ONFH patients was described as enlarged tongue body in pale or red, and a moist thick white tongue coating. The three categories of tongue manifestation may be applied to syndrome differentiation and identification staging of ONFH patients in combination with TCM theories. Despite the study's limitations, our findings contribute to the existing knowledge about tongue diagnosis, TCM syndrome differentiation and treatment options of ONFH patients. More quantitative analysis about tongue manifestation especially tongue body shape should be probed in the future, as well as its pathological mechanism.

## Funding

This study was supported by Beijing University of Chinese Medicine High‐level Talent Research Startup Project (2021‐XJ‐KYQD‐001), Investigation and analysis of the influence of smoking, drinking and scraping on tongue manifestation, National Natural Science Foundation of China (No.81873322, 82030122 and 81973888).

## Ethics Statement

Ethics was approved by the Institutional Review Board of Wangjing Hospital,

China Academy of Chinese Medical Sciences (No. 2008KT15). Informed consent was obtained from all participants included in the study.

## Author Contributions

Yan Jia and Jigao Sun are co‐first author, jointly drafted the manuscript and interpreted data. Zhaoxu Jia and Zhipeng Xue assisted with data acquisition and analysis. Weiheng Chen, Haijun He and Rongtian Wang provided funding and revised the manuscript.
